# Subjective social status, social network and health disparities: empirical evidence from Greece

**DOI:** 10.1186/s12939-017-0533-y

**Published:** 2017-02-27

**Authors:** Antonios Charonis, Ilias-Ioannis Kyriopoulos, Manos Spanakis, Dimitris Zavras, Kostas Athanasakis, Elpida Pavi, John Kyriopoulos

**Affiliations:** 1 MSD Greece, 63 Agiou Dimitriou Street, Alimos Athens, 17456 Greece; 2 London School of Economics and Political Science, Houghton Street, London, WC2A 2AE UK; 3 Department of Health Economics, National School of Public Health, 196 Alexandras Avenue, Athens, 11521 Greece

**Keywords:** Self-rated health, Subjective social status, Social network, Socioeconomic status, Ordinal logistic regression, Greece

## Abstract

**Background:**

Several studies suggest that socioeconomic status affects (SES) affects self-rated health (SRH), both in Greece and internationally. However, prior research mainly uses objective measures of SES, instead of subjective evaluations of individuals’ social status. Based on this, this paper aims to examine (a) the impact of the economic dowturn on SRH in Greece and (b) the relationship between subjective social status (SSS), social network and SRH.

**Methods:**

The descriptive analysis is based on four cross-sectional surveys conducted by the National School of Public Health, Athens, Greece (2002, 2006, 2011, 2015), while the data for the empirical investigation were derived from the 2015 survey (Health + Welfare Survey GR). The empirical strategy is based on an ordinal logistic regression model, aiming to examine how several variables affect SRH. Size of social network and SSS are among the independent variables employed for the empirical analysis

**Results:**

According to our findings, average SRH has deteriorated, and the percentage of the population that reports very good/good SRH has also decreased. Moreover, our empirical analysis suggests that age, existence of a chronic disease, size of social network and SSS affect SRH in Greece.

**Conclusion:**

Our findings are consistent with the existing literature and confirm a social gradient in health. According to our analysis, health disparities can be largely attributed to socioeconomic inequalities. The adverse economic climate has impact on socioeconomic differences which in turn affect health disparities. Based on these, policy initiatives are necessasy in order to mitigate the negative impact on health and the disparities caused by economic dowturn and the occuring socioeconomic inequalities.

## Background

The existence of a social gradient in health has been adequately documented by a series of studies [1, 2]. In this sense, socioeconomic status (SES) is widely acknowledged as one of the most significant and consistent predictors of health status [3]. Studies in both economics and epidemiology have noted and analyzed extensively the nexus between health and SES, whereas the direction of causality between the two variables is a topic of high importance and interest [4]. Generally, it has been consistently found that SES has an association with several health indicators and problems [5]. Additionally, a common topic in the literature relates to the underlying channels and mechanisms through which the main dimensions of SES can affect health.

Roughly, there are different pathways for establishing this relationship, as the main components of SES (income, occupation, education) affect health status in a different manner [6]. The link between subjective measures of SES and health has been widely documented in empirical research, whereas the existing literature has identified two plausible mechanisms, which could potentially explain the socioeconomic gradient in health [7]. The first interpretation relates to material deprivation, and the fact that lower socioeconomic position is linked with deteriorated access to products and services which -directly or indirectly- influence health, such as healthy food, housing conditions or medical care. According to the second mechanism, the socioeconomic gradient in health can be further explained on the basis of psychosocial factors and stress associated with living in an environment of relative socioeconomic disadvantage. These psychosocial factors could be either direct (i.e. allostatic load) or indirect (e.g. adoption of unhealthy behaviours due to stress, such as excess drinking and smoking). In this context, using subjective social status (SSS) as a proxy for SES could allow us to capture more comprehensive and dynamic attributes of socioeconomic position. Specifically, SSS goes beyond the objective indicators of SES and the ‘relative social standing’, since it incorporates not only the current SES, but also past assessments and future prospects. Therefore SSS also reflects attributes associated with social prestige, family wealth and resources and life chances, all of which could potentially influence health [8]. Moreover, SSS can better capture the second mechanism, which relates to psychological pathways. For instance, SSS reflects some feelings and perceptions associated with anxiety, stress and the sense of inequality in the case of low-SES individuals. These characteristics appear to affect health, and thus SSS can identify this mechanism more clearly compared to the objective SES indicators [9].

Self-rated health (SRH) is arguably one of the most common measures of health status in social science and epidemiological research [10]. Much has been written about the real association between SRH and objective health status; however SRH is regarded as a consistent and robust predictor of future mortality and morbidity [11]. According to the existing literature, SRH is affected by several socioeconomic, demographic, psychosocial and behavioral factors [12]. Additionally, it is partly influenced and determined by socioeconomic factors such as the socioeconomic position, social network and coherence, social capital, income distribution and others [13].

Prior research has mostly focused on the association between health and several objective measures and indicators of SES. The existing literature has highlighted an obvious connection between SRH and the SES of individuals [14, 15]. It has been extensively observed and examined that differences in the SES significantly alter the responses in SRH. Thus, individuals with a lower SES are more likely to have poorer SRH [16, 17].

Moreover, a growing body of literature attempts to find a relationship between subjective social status (SSS) and SRH. Specifically, several findings validate the hypothesis that subjective perceptions about the socioeconomic status influence SRH, controlling for several objective socioeconomic and demographic variables [18–20]. It is noteworthy that several studies indicate that SSS is a more precise and consistent predictor of health status, compared with the corresponding objective measures, such as income, education and occupation [21, 22].

However, apart from the SES per se, a growing body of evidence suggests that social ties and network essentially influence health. The theoretical foundations of this relationship come from the seminal contributions of Emile Durkheim and John Bowlby [17]. Generally, the link between social network and health is initially defined by the macro environment, which partly determines the structure of the social network, and several idiosyncratic characteristics and psychosocial factors (i.e. social engagement and support, social interaction, quality interpersonal relationships and others). These psychosocial parameters essentially affect health through several channels, including health-related behaviours, mental health and physical health [23].

The analysis focuses on the Greek population, a country well within its seventh year in recession and 5 years in deep austerity measures. Therefore the reference population is special and ‘unique’, given that such an economic scenario is unprecedented in the modern economic history of developed countries.

The main purpose of this paper is to investigate and analyze two topics. Firstly, it focuses on the effects of the economic downturn on the SRH of the Greek population, alongside with a comparison of different time periods, before and during the economic crisis (2002, 2006, 2011, and 2015). Secondly, it aims to examine the relationship between the SES and the SRH of the Greek population, and more specifically the extent to which SSS affects SRH. The analysis focuses on the population of Greece, a country well within its 7^th^ year in recession and 5 years in deep austerity measures

## Methods

### Data

The current study is a part of an ongoing health interview survey that started in 2002 and continues up to date. The present study undertakes a comparative and descriptive analysis of the data gathered on the previous national cross-sectional surveys (2002, 2006 and 2011) in order to signify the main differences that can be observed before and during the economic crisis. In the 2002 survey, mail questionnaires have been circulated, and 926 individuals have participated. In the survey of 2006, personal interviews were conducted with the participation of 4003 individuals, while in the survey of 2011, 6569 individuals have been interviewed through telephone. The 2015 sample consists of 2012 respondents, who answered a structured questionnaire via computer-assisted telephone interviews. The above surveys have been combined, and their data were pooled, giving information from 13,510 individuals in order to descriptively analyze the differences in SRH. It is noteworthy that all samples are representative and stratified according to the population characteristics (urbanization, gender, age).

The second part of the study is based solely on the gathered data of the national cross-sectional survey that has been conducted between the 14^th^ of December 2014 and the 20^th^ of January 2015 in Greece. A representative national sample has been chosen, stratified by age, gender, geographic regions and degree of urbanization. The sample consists of 2012 interviewees from the total of the Greek adult population. Using the 2015 survey, given that the response variable (SRH) is ordinal, the data have been analyzed through an ordinal logistic regression model, in order to empirically identify the factors affecting SRH.

### Variables

The dependent variable was SRH (1 = very bad, 2 = bad, 3 = fair, 4 = good, 5 = very good), and the independent variables employed for the empirical analysis were: (a) urbanization (rural/urban), (b) gender (male/female), (c) marital status (married/single/divorced/widowed), (d) number of family members, (e) existence of public health insurance (yes/no), (f) existence of private health insurance (yes/no), (g) age, (h) size of social network, (i) existence of chronic disease (yes/no) and (j) subjective social status (10-point ladder based).

### Subjective social status and self-rated health

In order to clearly represent the SES of individuals, a question analyzing the SSS has been used in the 2015 survey. SSS can be comprehended as the individual’s perception about his/her socioeconomic standing, by asking individuals to describe and rank their socioeconomic level in a 10-rung ladder. This ladder-based survey tool has been developed [24], in order to investigate potential causation between social characteristics and health disparities.

The question used in the survey was cited as; “*imagine a 10 step ladder representing the social status of the Greek public. At the top, step 10, you can found the individuals with the higher social status –* i.e. *with the higher income, the better education and the better occupation. At the bottom, step 1, you can found the individuals with the lower social status –* i.e. *with the lower income, lower educational level and the worst occupation or unemployed. In which step of this ladder you would place yourself*”. It is noteworthy that 124 out of the 2012 interviewees did not answer the aforementioned question. The social network was measured through a question that has been asked as: “*how many people do you feel close with you, so you can count on them*”.

Similarly, the survey took advantage of a 5-point survey tool to identify the SRH of the public. The above question was quoted as; “*how would you rate your health today?”* and the possible answers were *“very bad, bad, fair, good, very good*”. Out of the 2012 respondents only 2 individuals did not answer the above question.

## Results

The descriptive statistics of the aforementioned samples reveal that the vast majority of respondents perceive that they have a good/very good status. Specifically, the sum of the percentages of those who report good or very good health status is 72.3, 71.0, 68.8 and 69.5% in 2002, 2006, 2011 and 2015 respectively.

In the 2015 survey (see Table 1 for descriptive statistics), 2.3, 5.4 and 22.8% of the respondents rate their health as very bad, bad and fair respectively. Additionally, 40.7% of the interviewees assessed that they have good health, while the 28.8% of the sample answered that their health is very good.

**Table 1:** Descriptive statistics (2002, 2006, 2011 and 2015)

Variable	Year			
	2002 *N* = 926	2006 *N* = 4003	2011 *N* = 6569	2015 *N* = 2012
Self- rated health status				
Very bad	0.8%	2.2%	2.0%	2.3%
Bad	4.2%	6.1%	4.9%	5.4%
Average	21.7%	20.3%	24.2%	22.8
Good	43.7%	37.5%	41.3%	40.7%
Very good	25.5%	32.6%	27.5%	28.8%
Urbanization				
Urban	–	63.5%	73.9%	74.3%
Rural	–	36.5%	26.1%	25.7%
Gender				
Male	39.4%	49.3%	48%	47.6%
Female	58.3%	50.7%	52%	52.4%
Marital Status				
Married	–	59.4%	69.4%	63.4%
Single	–	29%	20.4	25%
Divorced	–	2.8%	2.9%	3.1%
Widow	–	7.6%	7.3%	8.5%
Number of family members^a^	–	–	2.99(2.12)	2.94(1.33)
Public health insurance				
Yes	–	95%	95.1%	91.9%
No	–	4.1%	4.6%	7.3%
Private health insurance				
Yes	–	11.9%	15.6%	13.2%
No	–	85.6%	83.6%	85.5%
Age				
18–24	2.9%	1.5%	6.7%	9.4%
25–39	24.9%	13.3%	25.1%	25.9%
40–54	32.4%	22.1%	28.1%	26.4%
55–64	17.7%	16.1%	17.6%	15.4%
65+	19.4%	46.4%	22.4%	22.9%
Existence of chronic disease				
Yes	–	36.4%	40.1%	42.1%
No	–	62.7%	59.9%	57.9%
Social network^b^				
0	–	–	–	3.4%
1–2	–	–	–	28.7%
3–5	–	–	–	46.6%
Over 6	–	–	–	21.2%
Self-rated SES				
Scale 1 – Lowest social status	–	–	–	4.3%
Scale 2	–	–	–	3.1%
Scale 3	–	–	–	5.6%
Scale 4	–	–	–	7.6%
Scale 5	–	–	–	25.7%
Scale 6	–	–	–	15.2%
Scale 7	–	–	–	17.4%
Scale 8	–	–	–	10.2%
Scale 9	–	–	–	2.7%
Scale 10 – Higher social status	–	–	–	2.0%

^a^Average figure of family members is showed (std deviation)

^b^Social network has been measured via the question “how many people do you feel close with so you can count on them?”

We also measured SRH for each respondent, based on a scale ranged between 0 and 100 (0 captures the lowest possible health status – 100 represents the highest one). Based on this variable, the average SRH has deteriorated in the last years. Specifically, in 2002 survey average SRH was 77.7, whereas it decreased to 76.7 in 2006. From then on, the corresponding size has decreased to 75.8 in 2011 and dropped further to 74.8 in 2015.

Table 1 presents the frequency of responses, including the SSS of the Greek population. Specifically, 70.2% of the population rates its social status between scale 4 and scale 7 of the 10-rung ladder in 2015. Therefore, most of the respondents consider themselves at a moderate social status level.

According to the empirical analysis, there is no statistically significant association between the SRH and urbanization, gender, marital status, the number of the family members and the type of health insurance. However, it is palpable that there is a statistically significant association between SRH and age, chronic disease status, social network and SSS. The detailed description of the empirical analysis is presented in Table 2.

**Table 2:** Ordinal logistic regression of self-rated health status on socio-demographic factors and subjective social status (2015)

Variable	OR	*P*	95% Confidence Interval
Age	0.74	<0.001	0.69–0.80
Existence of chronic disease^a^	4.30	<0.001	3.52–5.27
Social network	1.27	<0.001	1.13–1.42
Subjective social status	1.24	<0.001	1.18–1.29
/cut1	−1.10		(−1.75)–(−0.44)
/cut2	0.23		(−0.38)–(0.84)
/cut3	2.22		(1.61)–(2.83)
/cut4	4.44		(3.80)–(5.08)

^a^The reference category for existence of chronic disease is ‘yes’

More precisely the odds ratio for the age of the respondents is 0.74 (CI: 0.69–0.8); a fact that indicates that the individuals who belong in higher age groups have lower probability of rating their SRH in a better level. Furthermore, the odds ratio for the individuals who do not have a chronic disease is 4.40 (CI: 3.52–5.27), demonstrating that these individuals have a higher probability of rate their health status at a better level. Furthermore, the odds ratio for the size of the social network of the respondents is 1.27 (CI: 1.13–1.42), indicating that individuals who have more people to rely on have a higher probability to rate their health status in a better level. Finally, the odds ratio for the SSS is 1.24 (CI: 1.18–1.29). Therefore, individuals who rate themselves at a higher social status are more likely to rate their health status at a better level.

## Discussion and conclusion

In this study, we extend prior research on socioeconomic and demographic determinants of SRH in Greece [25, 26]. The main difference from previous work on this topic in Greece lies on the use of SSS instead of the conventional objective indicators of SES. To the best of our knowledge, this is the first study aiming to capture the SES by using a subjective indicator for the Greek population.

According to the results of the empirical analysis, SRH of the Greek public is associated with several determinants. As mentioned earlier, SRH can be regarded as a precise indicator that provides a good reflection of health status [8].

The findings suggest that age affects SRH, since older individuals are more likely to report poorer SRH. A reasonable explanation for this finding relates to the presence of multiple disease symptoms that are more prevalent to older individuals. Generally, and in accordance to the findings of the present study, the existing research suggests that the prevalence of poor SRH is better for older age groups [27, 28].

The empirical analysis indicates that the existence of a chronic disease is associated with higher odds of reporting poor SRH. Prior research has found that chronic patients are more likely to report poor SRH [29, 30]. However, according to the existing literature, different chronic diseases may have a different impact on SRH and the general health perceptions [31].

The aforementioned have also been found and analyzed extensively by several studies regarding the Greek population [11, 25, 26]. These studies have also noted that socioeconomic variables are statistically significant determinants of SRH.

Given that several studies indicate that SSS is a more consistent predictor of health status [17, 18], the importance of using an alternative subjective measure of SES is profound. The main advantage of our approach to SES is based on the claim that it is not merely the income level or the education that affects SRH, but also the general self-perception about the SES, the sense of belonging in a social class and the relative position in the social hierarchy [32]. Based on this, SSS can be considered as a broader concept than the objective measures of SES.

According to the empirical analysis, a higher perception about the social status is accompanied with higher odds of reporting very good SRH. Although this result is not surprising and seems quite reasonable, it can lead to different explanations regarding the relationship between SES and health disparities. Specifically, the role of SSS can provide a psychosocial aetiology regarding differences in SRH, instead of the material or objective differences in income, education and occupation [33].

It is noteworthy that a large body of evidence is in line with our findings regarding the relationship between SSS and SRH [18, 34]. Generally, SSS is considered as a statistically significant regressor, even if the health outcomes and measures change and after controlling for covariates [15].

According to our findings, individuals who have a larger social network are more likely to report better SRH levels. Generally, the relationship between social network and health is based on strong theoretical elements, which link macro-environment to the structure of social network, which in turn affects phychosocial mechanisms and health [23]. Specifically, several social and structural aspects of the macro-environment, (i.e. culture, inequality, poverty, discrimination, politics, labour market structure and economic performance) appear to determine the extent, structure and nature of social networks. Apart from the aforementioned ‘upstream’ link, Berkman et al. [23] mentioned that the size and structure of the social networks have impact on psychosocial mechanisms, such as (a) social support, (b) social influence, (c) social engagement, (d) interpersonal relationships, and (e) access to resources and material goods. The last link of this theoretical framework is based on the pathways from phychosocial aspects to health. Specifically, social networks (via psychosocial mechanisms described above) appear to affect health through three main pathways. The first relates to health behaviour, such as smoking, alcohol consumption, and exercise. Second, there is a psychological pathway, as social network is associated with self-esteem, sense of well-being and mental health [35]. Last, the literature has identified a link between social network and physiologic aspects. For instance, evidence suggests that social isolation is associated with lower immune function and cardiovascular activity [36, 37].

Apart from the theoretical basis, our results are consistent with the empirical literature on the relationship between social network and health status, using several different health indicators [38–41].

Generally, our findings suggest that inequalities in SRH can be associated with several reasons, including socioeconomic aspects, such as the SES and the size of social network. Given the existence of a social gradient in health, the current economic environment in Greece fosters socioeconomic inequalities, which in turn increase health disparities. It is noteworthy that several studies have indicated that the adverse economic climate has affected health status [26], utilization and access to healthcare [42, 43]. Taking into account the existing findings, as well as the ones presented in this study, a shift of the agenda towards social policy initiatives is essential in order to mitigate the adverse health effects and disparities caused by economic crisis and the occurring socioeconomic inequalities.

### Strengths and limitations

Despite there are some studies having analyzed similar topics, the present study makes several contributions to the literature for the factors affecting SRH. To our knowledge, this is the first study aiming to examine the relationship between social network and SRH in Greece. Moreover, this is the first empirical analysis that uses subjective measures to capture SES. Generally, there are several advantages for using SSS instead of objective measures of SES, which were briefly mentioned in this paper. Therefore, this study contributes to the existing literature on health status of Greek population (a) by introducing a new and more robust indicator for the SES and (b) by using variables in order to capture the effects of social network on health.

It is noteworthy that this study has some limitations, which should be acknowledged. First, the used dataset is based on a cross-sectional survey, and therefore it allows us to establish an association, but not a causal relationship. Second, our empirical analysis does not control for several variables, which may influence health. Third, we controlled for the size of social network, but not for alternative measures which potentially relate to social network, due to data availability issues.

## Abbreviations

SES: Socioeconomic status
SRH: Self-rated health
SSS: Subjective social status
